# Microflow imaging: New Doppler technology to detect low-grade inflammation in patients with arthritis

**DOI:** 10.1007/s00330-017-5016-4

**Published:** 2017-10-11

**Authors:** A. K. P. Lim, K. Satchithananda, E. A. Dick, S. Abraham, D. O. Cosgrove

**Affiliations:** 1 Department of Imaging, Imperial College Healthcare NHS Trust, Charing Cross Hospital, Fulham Palace Road, London, W6 8RF UK; 2 Department of Experimental Medicine and Therapeutics, Imperial College London, Hammersmith Hospital Campus, Du Cane Road, London, W12 0HS UK; 3 Digestive Diseases, Department of Surgery and Cancer, Imperial College London, QEQM, St. Mary’s Hospital, Praed Street W2, London, UK; 4 Department of Rheumatology and Medicine, NIHR/Wellcome Clinical Research Facility, Imperial College London, Hammersmith Hospital Campus, Du Cane Road, London, W12 OHS UK

**Keywords:** Ultrasound, Doppler, Arthritis, Inflammation, Tendinitis

## Abstract

**Aim:**

To assess the efficacy of microvascular imaging in detecting low-grade inflammation in arthritis compared with Power Doppler ultrasound (PDUS).

**Method and materials:**

Patients presenting for ultrasound with arthralgia were assessed with grey-scale, PDUS and Superb Microvascular Imaging (SMI). Videoclips were stored for analysis at a later date. Three musculoskeletal radiologists scored grey-scale changes, signal on PDUS and/or SMI within these joints. If a signal was detected on both PDUS and SMI, the readers graded the conspicuity of vascular signal from the two Doppler techniques using a visual analogue scale.

**Results:**

Eighty-three patients were recruited with 134 small joints assessed. Eighty-nine of these demonstrated vascular flow with both PD and SMI, whilst in five no flow was detected. In 40 joints, vascularity was detected with SMI but not with PDUS (p = 0.007). Out of the 89 joints with vascularity on both SMI and PDUS, 23 were rated as being equal; while SMI scored moderately or markedly better in 45 cases (p <0.001).

**Conclusion:**

SMI is a new Doppler technique that increases conspicuity of Doppler vascularity in symptomatic joints when compared to PDUS. This allows detection of low grade inflammation not visualised with Power Doppler in patients with arthritis.

***Key Points*:**

• *SMI detects vascularity with improved resolution and sensitivity compared to Power Doppler.*

• *SMI can detect low-grade inflammation not seen with Power Doppler.*

• *Earlier detection of active inflammation could have significant impact on treatment paradigms.*

**Electronic supplementary material:**

The online version of this article (doi:10.1007/s00330-017-5016-4) contains supplementary material, which is available to authorized users.

## Introduction

Ultrasound is a widely utilised, easily accessible, cross-sectional imaging modality that has the ability to assess the vascularity of lesions by using Doppler technology. It has many advantages over other cross-sectional modalities including ease of access, real-time imaging allowing dynamic assessment of the patient, and excellent sub-centimetre spatial resolution [1–5].

Ultrasound’s capability to visualise the microvasculature continues to improve, where Power and Colour Doppler ultrasound have already become a mainstay for a quick and non-invasive method of assessing the vascularity in tumours and tissue. The advent of microbubbles offers greater sensitivity and resolution of microvessels but requires an intravenous administration of contrast.

The presence of vascularity plays an important role in the assessment of patients with joint or tendon pain, particularly those with an established arthritis as this would denote active disease that may require a modification of treatment or involve a steroid injection. It has been widely accepted that Power Doppler is the standard of care to detect active synovitis in these patients [2–4].

A new Doppler technique developed by Toshiba Medical Systems termed ‘Superb Microvascular Imaging’ (SMI) purportedly allows better imaging of the microvasculature by employing an advanced Doppler algorithm without the need for contrast enhancement. This technology utilises a unique algorithm and filters that improve the detection of real flow while supressing bulk tissue motion more effectively than conventional methods. The result is that slower flow can be detected with better spatial resolution and improved sensitivity. This has been anecdotally reported to provide improved resolution when compared with current Power Doppler techniques (PDUS) [6–8].

The aim of this study was to therefore assess the efficacy of SMI in detecting low-grade inflammation in joints compared with the current ‘gold standard’ conventional Power Doppler ultrasound (PDUS).

## Materials and methods

### Patients and inclusion criteria

The study was approved by our institutional review board. Patients were recruited from a dedicated musculoskeletal (MSK) ultrasound rheumatology clinic between July 2013 and August 2015. They were all referred for arthralgia and to ascertain the presence of an active synovitis ultrasonically. Studies were only included for analysis if there were any one of the following abnormalities on their scan:

- Grey-scale changes of the joint including synovial hypertrophy, erosions or an effusion.
- Vascularity/signal seen with Power Doppler imaging
- Vascularity/signal seen with SMI.

The serum inflammatory markers erythrocyte sedimentation rate (ESR) and C-reactive protein (CRP) were also collated retrospectively in this cohort of patients.

In addition, the metacarpo-phalangeal joints of ten healthy normal volunteers were scanned as a control group

### Ultrasound scan technique

The ultrasound studies were performed by a MSK radiologist with more than 10 years experience of MSK ultrasound on an Aplio 500 scanner (Toshiba Medical Systems, Nasu, Japan). All scans were performed with an 18 mHZ probe and the joints were scanned in both the longitudinal and transverse planes with minimal pressure and ample gel as a stand off. The Power Doppler settings were set at 500–750 Hz and gain turned to just below noise to avoid significant artefact. SMI settings were standardised manufacturer recommendations. A spectral Doppler trace to confirm vascular flow was also obtained if it was difficult to distinguish between true signal and artefact, particularly if flow was seen with SMI or PDUS only.

Still images and videoclips of both the grey-scale images as well as Power Doppler and SMI of the affected joint or tendon were obtained and archived on the Hospital’s PACS (GE Medical systems, Milwaukee, WI, USA).

### Image analysis

These studies, both still images and videoclips, were then independently read by three MSK radiologists at a later date, blinded to the patients’ history, clinical examination and serum blood markers.

All readers were asked to score if there was vascular signal within the imaged joint, with PDUS, SMI or both.

If signal was detected on PDUS and SMI, the readers were also asked to score the conspicuity of PDUS or SMI using a four-point visual analogue scale comparing the two Doppler techniques based on sensitivity and resolution of the vessels visualised in the region of interest:

- 0 = No difference
- 1 = Mildly better (up to 25 % more vessels detected)
- 2 = Moderately better (25–50 % more vessels detected)
- 3 = Markedly better (>50 % of vessels detected)

For statistical analysis, the data collated above was treated as ordinal data, with one of seven possible categories (-3 to 3); where -3 strongly favours PDUS and 3 strongly favours SMI, while 0 = equipoise. The median of the scores for each joint provided by the readers was used for analysis.

#### Statistical analysis

Graphpad QuickCalcs 2015 and SPSS v22 were used for statistical analyses. Univariate analyses were performed using a Chi-squared test for categorical data, and a Kolmogorov-Smirnov test for categorical variables. *P* values <0.05 were considered significant. An intraclass correlation analysis (two-way random model) was carried out to test agreement between the three observers whose scores were treated as ordinal values.

## Results

### Patients

Eighty-three patients (9 males, 74 females), with a mean age of 44 years (range 29–81 years), were recruited prospectively. In total, there were 134 joints that met the inclusion criteria for analysis. In all cases, patients were symptomatic with joint pain and had a history of, or were being investigated for an arthropathy.

The diagnoses for this cohort of patients were osteoarthritis (n = 27), rheumatoid arthritis (n = 19), inflammatory arthritis (n = 16), psoriatic arthritis (n = 9) and no definitive diagnosis at time of study (n = 12)

### Joints scanned

The 134 joints scanned primarily included the radiocarpal, metacarpo-phalangeal, proximal inter-phalangeal and carpo-metacarpal joints. In five patients, the tarso-metatarsal joints, talo-navicular or tibio-talar joints were assessed, while in two patients the acromioclavicular joints were included in the analysis.

### PDUS versus SMI

Eighty-nine joints demonstrated vascular flow with both PDUS and SMI, while in five cases no flow was detected with either technique. In 40 joints, however, vascularity was detected with SMI but not with PDUS (Chi-squared (1, 40) = 7.41; p = 0.007) and in no case was a signal detected with PDUS but not SMI. These data are summarised in Table 1.

**Table 1:** This 2 × 2 table shows the number of joints where signals were seen with either SMI and/or PDUS or neither. Of note is the group of 40 cases where signal was only detected on SMI but not PDUS [Chi-squared (1, 40); p = 0.007]

N =134	Signal on SMI	No signal on SMI
Signal on PDUS	89	0
No signal on PDUS	40	5

**Fig. 1:**
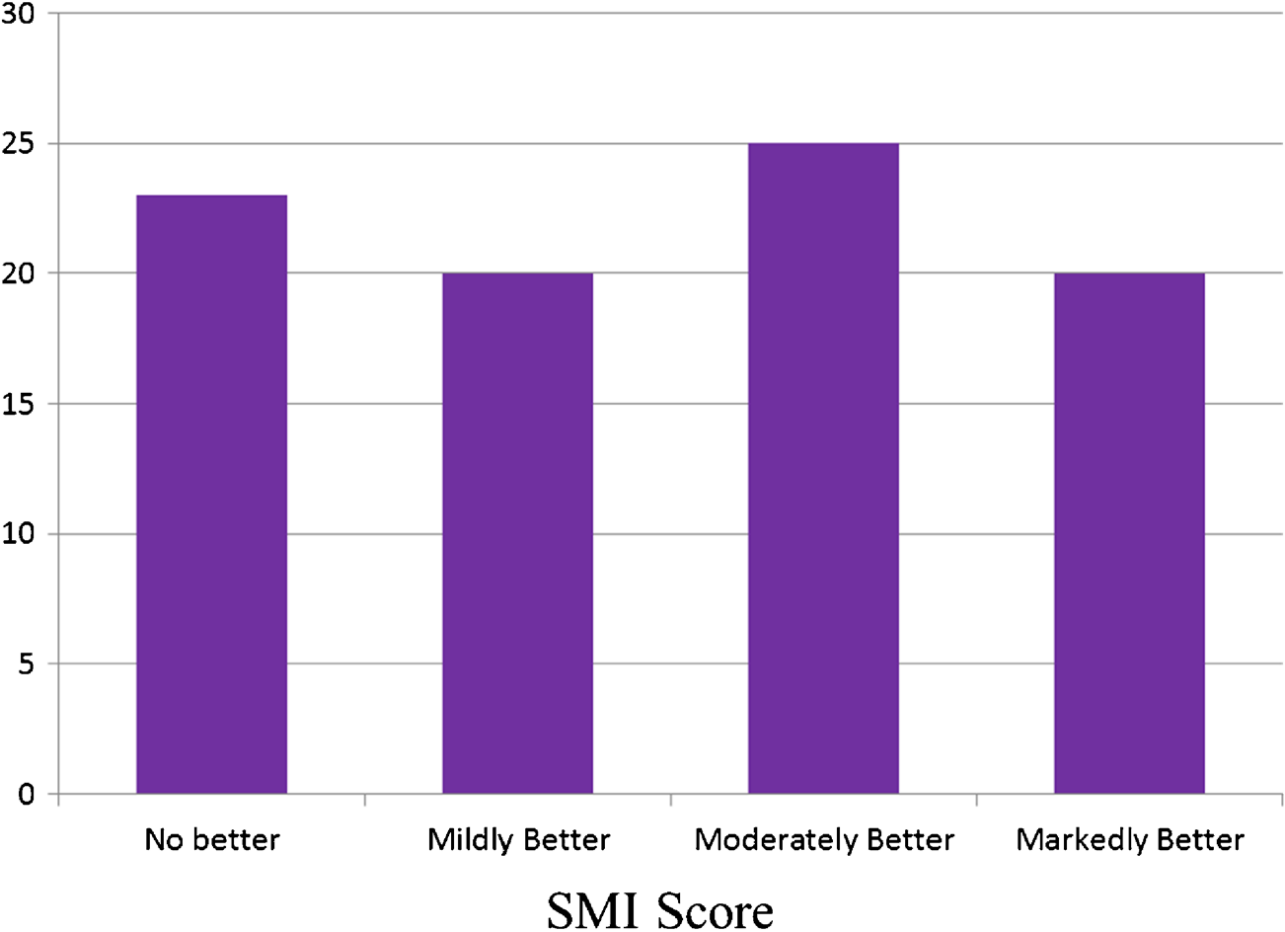
This graph shows the median visual analogue scores of the readers in cases where signal was seen on both Superb Microvascular Imaging (SMI) and Power Doppler ultrasound (PDUS). In all cases bar one, the readers were in agreement that in the majority SMI was better than PDUS (Kolmogorov-Smirnov test: p<0.001 for all three readers). In the single case where PDUS was better, the readers scored the conspicuity of PDUS to be mildly better than SMI

Out of the 89 joints with vascularity detected on SMI and PDUS, 23 showed no difference in conspicuity between the two techniques. In only one case was the PDUS score better than SMI for conspicuity. In 65 joints, SMI scored better than PDUS for conspicuity; in 45 of these the conspicuity difference was moderate or markedly better for SMI (one-sample binomial test: p<0.001). The relative conspicuity values for all three readers were normally distributed (mean ± standard deviation): Reader 1 (2.1 ± 1.55), Reader 2 (1.92 ± 1.57) and Reader 3 (2.23 ± 1.47) (Kolmogorov-Smirnov test: p<0.001 for all three readers). Figure 1 is a graph showing the distribution of scores of the readers comparing SMI to PDUS in those cases where signal was detected on both Doppler techniques.

**Fig. 2:**
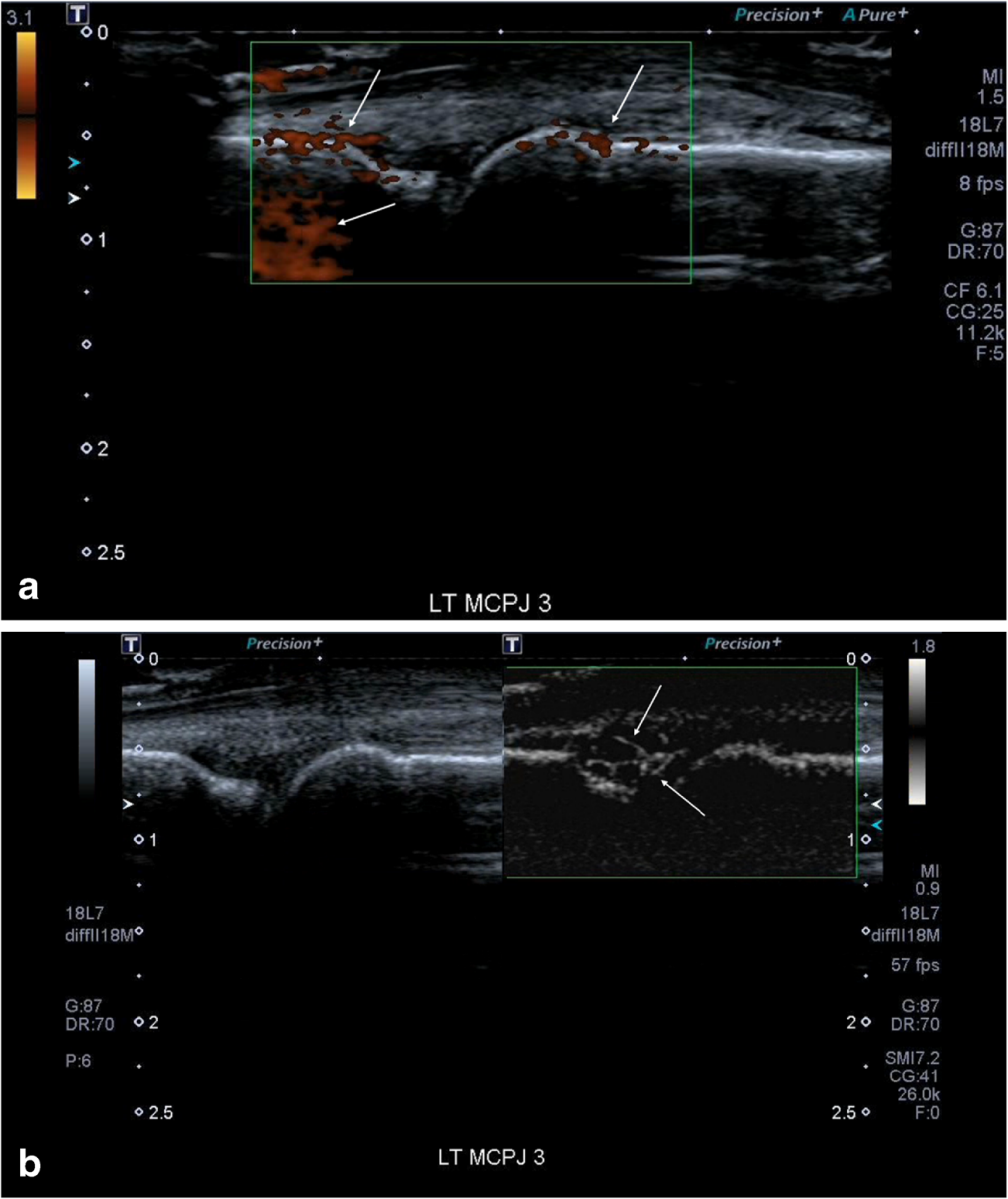
(**a**) This shows an image of a metacarpophalangeal joint (MCPJ) in a patient with an inflammatory arthritis and a symptomatic joint. Even with the Power Doppler gain turned right up and a low scale, resulting in much noise (white arrows), there remains no vascular signal within the joint to support an active synovitis. (**b**) There is much vascularity seen with Superb Microvascular Imaging (SMI) within the joint with fine spatial resolution (arrows). This would therefore denote an active synovitis in keeping with the patient’s symptoms and also elevated erythrocyte sedimentation rate (ESR) and C-reactive protein (CRP)

**Fig. 3:**
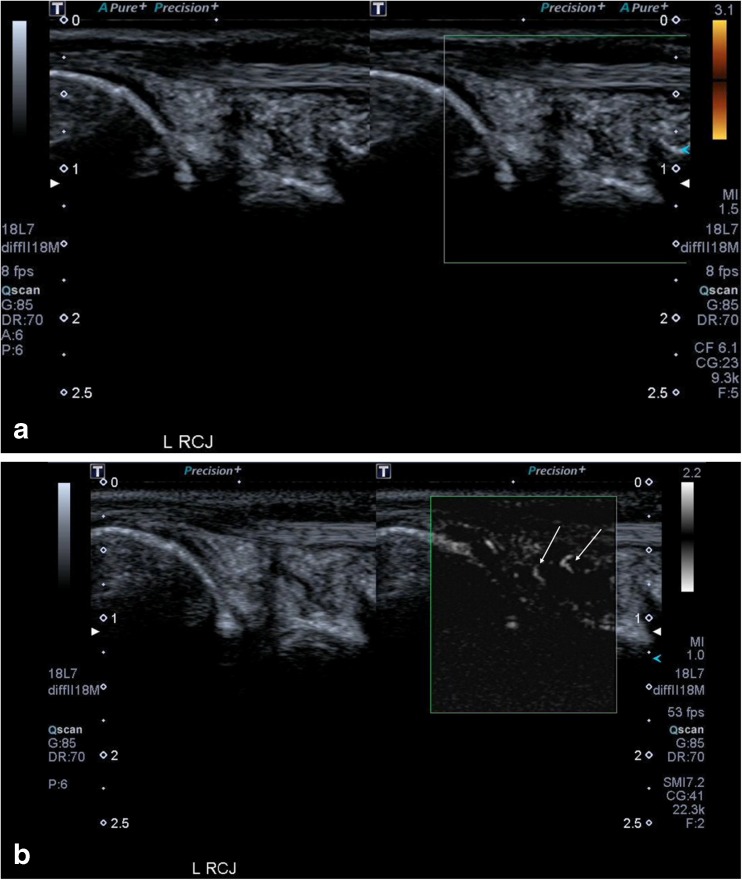
(**a**) These images show synovial hypertrophy on the grey-scale image of the radiocarpal joint (RCJ) in a patient with rheumatoid arthritis. In the dual image the right side of the split screen shows no detectable vascular flow within the thickened synovium on Power Doppler ultrasound (PDUS). (**b**) This second pair of images shows that there is clear neovascularity within the joint (arrows) on Superb Microvascular Imaging (SMI) indicating active inflammation, which was not evident on PDUS. The increased sensitivity and spatial resolution of SMI is much better appreciated on videoclips rather than still images and these have been included as supplementary material for this patient

**Fig. 4:**
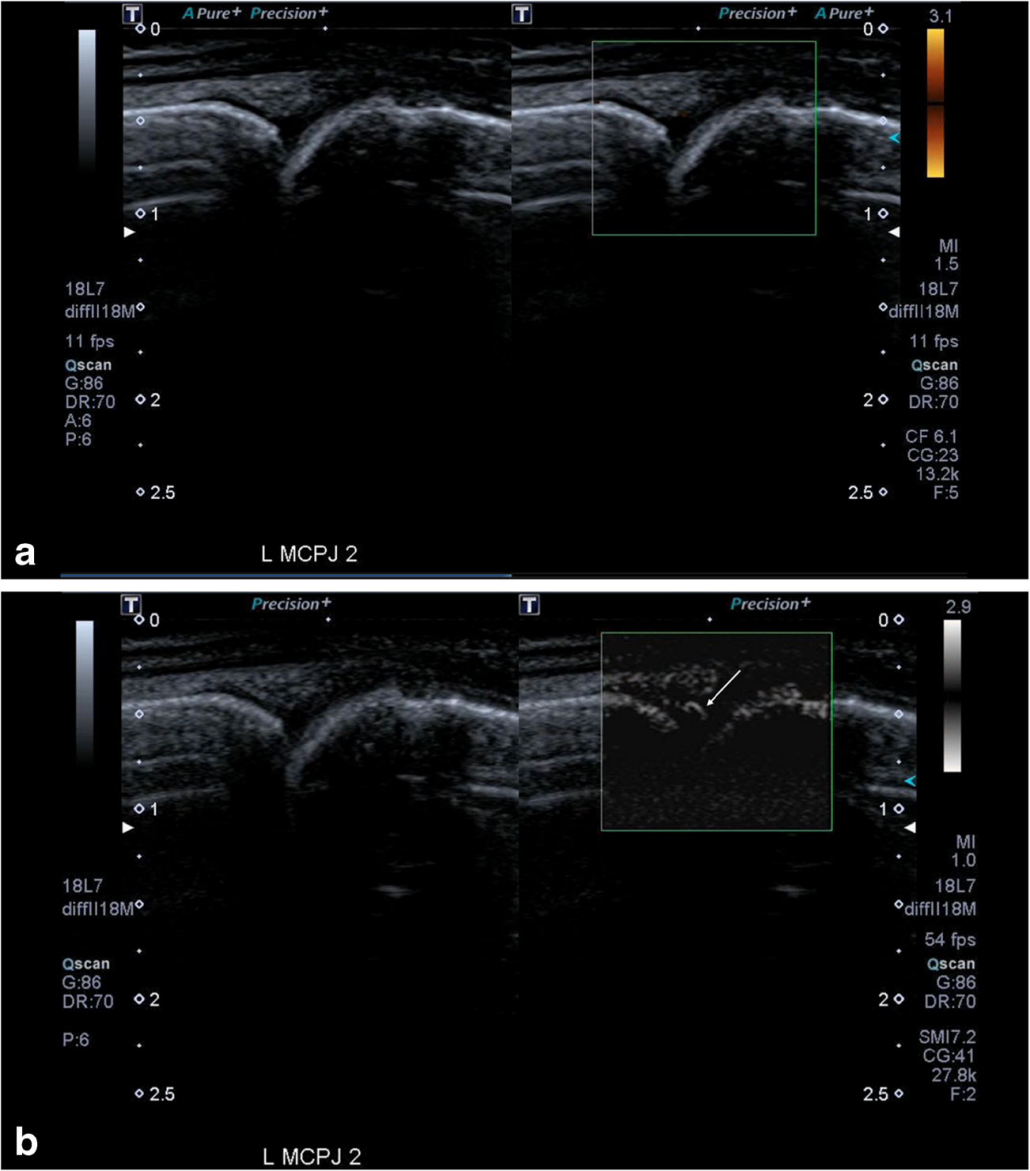
(**a**) These images show a relatively normal appearing joint on the grey-scale image of the metacarpophalangeal joint (MCPJ) of the left index finger, which was tender in this patient with rheumatoid arthritis. There is also no signal detected on Power Doppler ultrasound (PDUS). (**b**) The corresponding paired Superb Microvascular Imaging (SMI) images show that there is clear neovascularity seen within the joint (arrows) indicating active inflammation. This image highlights the resolution and flow within very small vessels that can be detected with SMI

Figure 2a and b illustrate a metacarpophalangeal joint of a patient with an inflammatory arthritis where Doppler signal was only seen on SMI and not with PDUS, where the Power Doppler gain has been increased such that there is much noise but there remained no signal within the joint. Paired Figs. 3a and b, and 4a and b, illustrate the improved sensitivity and spatial resolution of SMI compared with PDUS in detecting low-grade inflammation in a radiocarpal joint of a patient with rheumatoid arthritis. These are even more apparent with the paired short videoclip attached.

In all patients with positive findings on PDUS or SMI in symptomatic joints, there was no vascularity on SMI or PDUS in asymptomatic joints.

### Interobserver variation

The intraclass correlation coefficient was highly significant (0.935, p<0.001). A value >0.75 indicates excellent correlation.

### Serum markers of inflammation

All patients were symptomatic with arthralgia thus warranting an ultrasonographic assessment to detect active inflammation. In the 40 patients where vascular signals within the joints were detected on SMI only but not PDUS, 25 also had elevated ESR (>12 mm/h) or CRP (>5 mg/L) with a mean ESR of (32.7 ± 12.7) mm/h and mean CRP of (9.0 ± 18.6) mg/L. In ten patients, the serum markers were normal and in five these were not available.

The average interval between the serum markers and ultrasound study was 29.6 days (range: 0–91 days; median: 27.5 days).

### Healthy volunteers

The metacarpophalangeal joints of a small cohort of ten normal volunteers (age range 26–35 years; total number of joints = 100) did not reveal any vascularity in these joints on either PDUS or SMI.

## Discussion

It is widely accepted that the capability to detect pathological flow within MSK soft tissues with Doppler ultrasound denotes the presence of local active inflammation [9–13].

Advances in ultrasound Doppler technology continue to improve sensitivity and spatial resolution, and include new ultrafast Doppler techniques and SMI [6–8]. Preliminary data suggest that the use of these technologies, particularly SMI, improves resolution and sensitivity when compared with standard PDUS [7, 8]. This is made possible by state-of-the art filtering algorithms, which unlike conventional Doppler filters, manage to remove only noise from the image while preserving signal from the slow flowing, very small vessels. This is can be achieved by adding temporal to spatial filtering but is only possible with coding using a Graphic Processing Unit (GPU).

To our knowledge this is one of the first original research articles reporting the clinical utility of this new Doppler technology. The main aim of this study was to ascertain whether this improvement (i.e. SMI) had greater diagnostic conspicuity than PDUS in detecting vascularity in symptomatically inflamed joints, thereby also indirectly assessing the clinical value with respect to MSK imaging where Doppler modalities are currently considered an integral part of the global sonographic assessment of joints and tendons.

In our study, the key finding was that in 40 out of the total of 134 (30 %) joints analysed, Doppler signals were detected using SMI but not with PDUS. This correlated with the site of symptoms. Since all non-symptomatic joints in the same patient did NOT exhibit SMI Doppler flow, we considered that this represented a true-positive finding rather than a false-positive finding. Further evidence supporting the SMI findings as true positives includes the excellent interobserver agreement between three MSK radiologists blinded to the clinical findings and the fact that in this subset of patients the serum inflammatory markers were elevated in 25/40 (70 %) of these patients. We infer therefore that SMI depicts low-grade, subclinical inflammatory activity that cannot be detected by PDUS.

The results from our study also confirm the previous anecdotal evidence of improved conspicuity of SMI when compared with PDUS in joints. In the majority of the cases where signals were seen on both PDUS and SMI, the three readers scored SMI as providing better conspicuity than PDUS (see Fig. 1). Excellent interobserver agreement between all three readers again strengthens the validity of these findings. In addition, there have also been a couple of recent studies that have reported that SMI provides improved delineation of the microvasculature in breast tumours when compared with colour or Power Doppler [9, 10]. This lends further support to the findings of our SMI study in joints and that the signals detected are real.

The advent of SMI would appear to be of great clinical significance where currently PDUS is the accepted gold standard for detecting active inflammation in the setting of a swollen or tender joint [10–13]. Several previous studies have shown that, although the presence of vascular flow on PDUS indicates active inflammation, the lack of signal cannot reliably exclude disease activity [14–17]. This highlights the unmet need of inflammatory arthritis and the inability to detect subclinical inflammation, which can lead to joint erosion if the active inflammation is not halted.

SMI may be able to identify a subsection of patients with inflammatory disease who do not have neovascularity detected by PDUS, yet who do have inflammatory arthritis. In these patients it has been postulated that standard PDUS may not be sensitive enough to delineate very slow velocity blood vessels in the microvasculature of the synovium.

In addition, there have also been many studies reporting the utility of Doppler ultrasound as a biomarker of disease response to treatment in patients with arthritis, and we postulate that with future longitudinal studies, SMI may become a biomarker of disease response to treatment in patients with arthritis, similar to PDUS currently [18]. A further advantage of SMI is that it is non-invasive, which is advantageous compared to contrast-enhanced ultrasound techniques (CEUS) where previous studies have reported improved detection of inflammation in joints with just PDUS alone [19].

This is the first study reporting the improved SMI findings in patients with arthritis utilising this state-of the art Doppler technology when compared with standard-of-care PDUS. Larger scale multicentre studies are now needed to provide validation of this pilot study.

## Limitations

One of the limitations of this study is the lack of an age-matched formal control group; however, the metacarpophalangeal joints of the small cohort of ten normal volunteers did not reveal any vascularity on either PDUS or SMI. Nonetheless, a patient’s asymptomatic joint acted as a control within the cohort. No asymptomatic joints demonstrated vascularity on SMI or PD in our patient population.

Another possible limitation is a lack of quantitative analysis of the data but this is currently not possible owing to the unavailability of such software and quantifying the vascularity of a three-dimensional structure still remains challenging. However, there was excellent interobserver agreement based on our visual analogue scale of vascular conspicuity.

## Conclusion

SMI is a new Doppler technique that increases conspicuity of Doppler vascularity in symptomatic joints when compared to PDUS. This allows detection of low-grade inflammation not visualised with Power Doppler in patients with arthritis.

## Electronic supplementary material

ESM 1 (MPEG 846 kb)

ESM 2 (MPEG 2809 kb)

## Abbreviations

CEUS: Contrast-enhanced ultrasound
CRP: C-Reactive protein
ESR: Erythrocyte sedimentation rate
MCPJ: Metacarpophalangeal joint
MSK: Musculoskeletal
PACS: Picture Archiving and Communications System
PD: Power Doppler
PDUS: Power Doppler ultrasound
RCJ: Radiocarpal joint
SMI: Superb Microvascular Imaging

## References

[1] Wakefield RJ, Brown AK, O’Connor PJ, Emery P (2003). Power Doppler sonography: improving disease activity assessment in inflammatory musculoskeletal disease. Arthritis Rheum.

[2] Larche MJ, Seymour M, Lim AK (2010). Quantitative power Doppler ultrasonography is a sensitive measure of metacarpophalangeal joint synovial vascularity in rheumatoid arthritis and declines significantly following a 2-week course of oral low-dose corticosteroids. J Rheumatol.

[3] Szkudlarek M, Narvestad E, Klarlund M, Court-Payen M, Thomsen HS, Østergaard M (2004). Ultrasonography of the metatarsophalangeal joints in rheumatoid arthritis: comparison with magnetic resonance imaging, conventional radiography, and clinical examination. Arthritis Rheum.

[4] Wakefield RJ, Balint PV, Szkudlarek M, Filippucci E, Backhaus M, D’Agostino MA, Sanchez EN, Iagnocco A, Schmidt WA, Bruyn GA (2005). Musculoskeletal ultrasound including definitions for ultrasonographic pathology. J Rheumatol.

[5] Torp-Pedersen ST, Terslev L (2008). Settings and artefacts relevant in colour/power Doppler ultrasound in rheumatology. Ann Rheum Dis.

[6] Demene C, Deffieux T, Pernot M, Osmanski BF, Biran V, Franqui S, et al. (2015) Spatiotemporal clutter filtering of ultrafast ultrasound data highly increases Doppler and Ultrasound sensitivity. IEEE Trans Med Imaging 10.1109/TMI.2015.2428634 25955583

[7] Lim AK (2014) The Clinical utility of Superb Microvascular Imaging (SMI) for Assessing Musculoskeletal Inflammation. Toshiba Medical Systems, White paper

[8] Hata J (2014) Seeing the unseen. New Techniques in Vascular imaging: Superb Microvascular Imaging. Toshiba Medical Systems, White paper

[9] Xiao XY, Chen X, Guan XF, Wu H, Qin W, Luo BM (2016). Superb microvascular imaging in diagnosis of breast lesions: a comparative study with contrast-enhanced ultrasonographic microvascular imaging. Br J Radiol.

[10] Park AY, Seo BK, Cha SH, Yeom SK, Lee SW, Chung HH (2016). An Innovative Ultrasound Technique for Evaluation of Tumor Vascularity in Breast Cancers: Superb Micro-Vascular Imaging. J Breast Cancer.

[11] Koski JM, Saarakkala S, Helle M (2006). Power Doppler ultrasonography and synovitis: correlating ultrasound imaging with histopathological findings and evaluating the performance of ultrasound equipments. Ann Rheum Dis.

[12] Rees JD, Pilcher J, Heron C, Kiely PD (2007). A comparison of clinical vs ultrasound determined synovitis in rheumatoid arthritis utilizing gray-scale, power Doppler and the intravenous microbubble contrast agent ‘SonoVue’. Rheumatology.

[13] Porta F, Radunovic G, Vlad V (2012). The role of Doppler ultrasound in rheumatic diseases. Rheumatology (Oxford).

[14] Naredo E, Collado P, Cruz A (2007). Longitudinal power Doppler ultrasonographic assessment of joint inflammatory activity in early rheumatoid arthritis: predictive value in disease activity and radiologic progression. Arthritis Rheum.

[15] Naredo E, Rodriguez M, Campos C et al (2008) Validity, reproducibility, and responsiveness of a twelve-joint simplified power doppler ultrasonographic assessment of joint inflammation in rheumatoid arthritis. Arthritis Rheum:59515–59522 10.1002/art.23529 18383408

[16] Scire CA, Montecucco C, Codullo V (2009). Ultrasonographic evaluation of joint involvement in early rheumatoid arthritis in clinical remission: power Doppler signal predicts short-term relapse. Rheumatology.

[17] Brown AK, Conaghan PG, Karim Z (2008). An explanation for the apparent dissociation between clinical remission and continued structural deterioration in rheumatoid arthritis. Arthritis Rheum.

[18] Sreerangiah D, Grayer M, Fisher BA, Ho M, Abraham S, Taylor PC (2016) Quantitative power Doppler ultrasound measures of peripheral joint synovitis in poor prognosis early rheumatoid predict radiographic progression. Rheumatology (Oxford) 55(1):89–93 10.1093/rheumatology/kev305 PMC5854030 26316580

[19] Platzgummer H, Schueller G, Grisar J (2009). Quantification of synovitis in rheumatoid arthritis: do we really need quantitative measurement of contrast-enhanced ultrasound?. Eur J Radiol.

